# Cancer pharmacogenetics

**DOI:** 10.1038/sj.bjc.6601487

**Published:** 2004-01-06

**Authors:** S Marsh, H L McLeod

**Affiliations:** 1 Department of Medicine, Washington University School of Medicine, 660 South Euclid Ave, Campus Box 8069, the Siteman Cancer Center, and the CREATE Pharmacogenetic Research Network, St Louis, MO 63110-1093, USA

**Keywords:** pharmacogenetics, pharmacogenomics, polymorphism

## Abstract

The large number of active combination chemotherapy regimens for most cancers has led to the need for better information to guide the ‘standard’ treatment for each patient. In an attempt to individualise therapy, pharmacogenetics and pharmacogenomics (a polygenic approach to pharmacogenetic studies) encompass the search for answers to the hereditary basis for interindividual differences in drug response. This review will focus on the results of studies assessing the effects of polymorphisms in drug-metabolising enzymes and drug targets on the toxicity and response to commonly used chemotherapy drugs. In addition, the need for polygenic pharmacogenomic strategies to identify patients at risk for adverse drug reactions will be highlighted.

In the UK it has been estimated that approximately 7% of patients are affected by adverse drug reactions (ADRs). Indeed, one out of 10 of all NHS bed days are used by patients with ADRs. These ADRs result in the need for the equivalent of 15–20 400-bed hospitals and cost about £380 million a year. ADRs due to cancer chemotherapy are estimated to increase the overall hospital costs by 1.9% and drug costs by 15% (Wiffen *et al*, 2002). Clearly, the current regimen of ‘one dose fits all’ for chemotherapy treatment is not ideal for patients and is not cost effective for the health service. In an attempt to individualise therapy, pharmacogenetics and pharmacogenomics (a polygenic approach to pharmacogenetic studies) encompass the search for answers to the hereditary basis for interindividual differences in drug response (Evans and McLeod, 2003).

In addition to environmental influences, variation in the genetic constitution between individuals will have a major impact on drug activity. Single-nucleotide polymorphisms (SNPs) account for over 90% of genetic variation in the human genome. The remainder of the variation is caused by insertions and deletions (indels), tandem repeats and microsatellites. With the completion of the human genome project, there has been an explosion in the discovery, characterisation and validation of genetic variation. Over 1.42 million SNPs were initially identified through the human genome project (Sachidanandam *et al*, 2001), and a goldmine of SNP information is now readily accessible via publicly available databases (Marsh *et al*, 2002). In addition, affordable, high throughput genotyping technologies are now available, including Pyrosequencing, FP-TDI, MALDI-TOF and SNP chips, making pretreatment genotyping a real possibility. A comprehensive review of the technology available to pharmacogenetics research is beyond the scope of this minireview; however, there are many excellent reviews describing these applications (Kwok, 2001; Shi, 2001; Syvanen, 2001; Freimuth *et al*, 2003).

Pharmacogenomics includes studies of variations in germline DNA, somatic mutations and variations in RNA expression (Watters and McLeod, 2003). This minireview will focus on the impact of germline polymorphisms, highlighting their effects on the activity and response to commonly used chemotherapy drugs such as mercaptopurine, 5-fluorouracil, cyclophosphamide, platinum agents and camptothecins.

## THIOPURINE METHYLTRANSFERASE (TPMT)

Perhaps the most compelling evidence for the utility of pharmacogenomic strategies to identify patients at risk for adverse drug reactions comes from genetic polymorphisms in the gene (TPMT). Mercaptopurine is a commonly used treatment for childhood acute lymphocytic leukaemia. Thiopurine methyltransferase (TPMT) methylates mercaptopurine, reducing its bioavailability for conversion into thioguanine nucleotides (TGN), the cytotoxic form of the drug. Approximately 10% of patients have an intermediate enzyme activity and 0.3% are deficient for TPMT activity. Intermediate-activity patients have a greater incidence of thiopurine toxicity, whereas TPMT-deficient patients have severe or fatal toxicity from mercaptopurine therapy. To date, at least 10 variations in the TPMT gene have been associated with low TPMT enzyme activity (McLeod and Siva, 2002). Three of these variants (TPMT^*^2, TPMT^*^3A and TPMT^*^3C) account for up to 95% of low TPMT activity phenotypes. Patients heterozygous for these alleles have intermediate TPMT levels and tolerate approximately 65% of standard mercaptopurine dosage (Relling *et al*, 1999b). Patients homozygous for the variant TPMT alleles are at high risk for severe, sometimes life-threatening toxicity, requiring significant reductions in drug doses (one out of 10 to one out of 15 of the standard dose). Patients requiring dose reduction because of variant TPMT alleles have similar or superior survival compared to patients with the wild-type allele (Relling *et al*, 1999a). TPMT^*^3A is the most common allele in Caucasian populations, with a frequency between 3.2 and 5.7%. TPMT^*^2 and TPMT^*^3C alleles are present in 0.2–0.8% of Caucasians. A significant variation in TPMT allele frequencies is seen among different world populations. TPMT^*^3A is the only variation found in Southwest Asians (1%), whereas all variant alleles in African populations are TPMT^*^3C (5.4–7.6%) (McLeod and Siva, 2002). Pretreatment knowledge of a patient's TPMT genotype status is now being used in major centres for dose optimisation, in order to reduce prospectively the likelihood of adverse drug reactions in children with ALL.

## DIHYDROPYRIMIDINE DEHYDROGENASE (DPYD)

5-Fluorouracil (5FU) is one of the most commonly administered chemotherapy agents, used in combination therapy for treatment of colorectal, breast, head/neck and other solid tumours. Over 80% of 5-FU is inactivated by dihydropyrimidine dehydrogenase (DPD). Decreased DPD activity is associated with >four-fold risk of severe or fatal toxicity from standard doses of 5FU (Van Kuilenburg *et al*, 2002). To date, at least 20 polymorphisms in the DPD gene (DPYD) have been described (McLeod *et al*, 1998). However, many of these polymorphisms have not been definitively associated with altered DPD activity, and not all toxicity to 5FU from reduced DPD activity can be explained by the currently known DPYD polymorphisms. The most consistent data are for the allele DPYD^*^2A, a G>A splice site transition that causes skipping of exon 14. Patients heterozygous for this polymorphism have low DPD activity and toxicity to 5FU (Wei *et al*, 1996). However, the allele frequency for DPYD^*^2A in control populations appears to occur at a low frequency (approximately one out of 135 in Caucasians) (McLeod *et al*, 1998) and a more complete genotype–phenotype relationship study remains to be completed. Currently, the apparently high false-negative rate for DPYD as a predictor for severe 5FU toxicity (Collie-Duguid *et al*, 2000) restricts the testing of DPYD^*^2A to either research studies or as a component of a panel of oncology-related pharmacogenetic markers.

## UDP-GLUCURONOSYLTRANSFERASE 1A1 (UGT1A1)

Irinotecan, a camptothecin analogue, is used to treat colorectal, lung and other solid tumours. 5FU/irinotecan combination therapy is a common front-line therapy for colorectal cancer. The active form of irinotecan, SN38, can be inactivated through glucuronidation by a member of the UDP-glucuronosyltransferase family. A dinucleotide repeat in a TATA box in the UGT1A1 promoter results in altered UGT1A1 activity (Monaghan *et al*, 1996). Reduced UGT1A1 is linked to a high risk (approximately four-fold) of severe toxicity from irinotecan treatment, including dose-limiting diarrhoea and neutropenia (Wasserman *et al*, 1997). The variable number of TA repeats ranges from five to eight copies, six TA repeats represent the most common allele, with up to 33% in Caucasians having a variant allele containing seven repeats (UGT1A1^*^28) (Iyer *et al*, 1999). Significant associations between patients with the UGT1A1^*^28 allele and reduced UGT1A1 expression, and, consequently, reduced SN38 glucuronidation have been shown in several studies (Ando *et al*, 1998; Iyer *et al*, 1999; Iyer *et al*, 2002). Assessment of the presence of the UGT1A1^*^28 allele in patients prior to irinotecan treatment may predict individuals at risk for severe toxicity from irinotecan, allowing the selection of lower doses or alternative therapies.

## GLUTATHIONE *S*-TRANSFERASE P1 (GSTP1)

Glutathiones play a role in detoxifying, and consequently protecting cells from alkylating agents and products of reactive oxidation. The pi-class of human (GSTP1) has been found to catalyse glutathione conjugation of reactive metabolites from cyclophosphamide, a drug commonly used in the treatment of breast cancer and other solid tumours. In addition, GSTP1 is known to detoxify platinum compounds, including oxaliplatin, a relatively new chemotherapy drug used in combination with 5FU for the treatment of advanced colorectal cancer.

A SNP causes an isoleucine to valine substitution at amino-acid codon 105 (I105V) in the GSTP1 gene. The valine allele occurs at a frequency of 33% in Caucasian populations, and is associated with reduced GSTP1 activity compared to the isoleucine allele (Watson *et al*, 1998). This SNP has been correlated with response to cyclophosphamide chemotherapy treatment in breast cancer patients (Sweeney *et al*, 2000). In all, 240 patients treated with cyclophosphamide were characterised for the GSTP1 I105V SNP. Patients homozygous for the valine (low activity) allele had a 0.3 hazard ratio (95% confidence interval 0.1–1.0) for overall survival compared to patients homozygous for the isoleucine (high activity) allele. Patients heterozygous for the SNP had an intermediate hazard ratio for overall survival (0.8; 95% confidence interval 0.5–1.3) (Sweeney *et al*, 2000). In addition, a study of 107 patients with advanced colorectal cancer treated with a combination of 5FU and oxaliplatin were assessed for the GSTP1 I105V SNP (Stoehlmacher *et al*, 2002). Patients homozygous for the valine allele had a median of 24.9 months survival, compared to 7.9 months for patients homozygous for the isoleucine allele (*P*<0.001) (Stoehlmacher *et al*, 2002).

Currently, studies are mainly focused on the effect of SNPs in GSTP1 on the risk of cancer. Further research on the association of GSTP1 SNPs with response to alkylating agents and platinum drugs will provide information on the usefulness of prescreening patients for GSTP1 genotypes prior to treatment. In addition, genetic polymorphism in other genes involved in detoxification and repair have been associated with response or survival after platinum-containing chemotherapy (Park *et al*, 2001; Stoehlmacher *et al*, 2002), providing additional avenues for pharmacogenetic strategies to individualise therapy.

## THYMIDYLATE SYNTHASE (TYMS)

So far, this minireview has focused on the effects of polymorphisms in drug-metabolising enzymes in response to a range of chemotherapy drugs. Polymorphisms in drug targets are also an important area for pharmacogenetic studies, as overexpression or underexpression of drug targets could lead to resistance or toxicity to standard chemotherapy regimens.

The main target for 5-FU is thymidylate synthase (TS). Thymidylate synthase, together with a methyl cofactor, catalyses the methylation of dUMP to dTMP. The 5FU metabolite FdUMP forms a stable ternary complex with TS and the methyl cofactor, blocking the production of dTMP and ultimately inhibiting DNA synthesis. The overexpression of TS has been linked to resistance to 5-FU and other TS inhibitors, such as raltitrexed. Three polymorphisms have been described in the TS gene (TYMS). A polymorphic 28 bp tandem repeat in the promoter enhancer region (TSER) has been extensively characterised in multiple world populations (Marsh and McLeod, 2001a). The polymorphism varies from two (TSER^*^2) to nine (TSER^*^9) copies of the tandem repeat, with TSER^*^2 and TSER^*^3 being the most common alleles. The higher numbers of repeats are mainly found in African populations (Marsh *et al*, 2000), and their roles in TS expression are currently unknown. *In vitro* studies have demonstrated that TSER^*^3 has a higher TS expression than TSER^*^2 (Horie *et al*, 1995). In addition, small studies in cancer patients treated with 5FU have shown a correlation between TSER^*^3 and higher free TS protein levels (Kawakami *et al*, 1999), TSER^*^3 and reduced downstaging in rectal cancer (Villafranca *et al*, 2001) and TSER^*^3 and a lower response rate to 5FU treatment (Marsh *et al*, 2001b). In 2001, Villafranca and colleagues studied the TSER in 65 rectal cancer patients treated with chemoradiation. Over 60% of patients with at least one TSER^*^2 allele had downstaging of their rectal tumours (a marker of positive response to treatment), whereas only 22% of patients homozygous for TSER^*^3 had tumour downstaging (*P*=0.002) (Villafranca *et al*, 2001). Large-scale patient studies are now underway to better define the role of the TSER polymorphism to outcome from 5FU chemotherapy. However, a greater degree of resolution between ‘good’ and ‘poor’ outcome is needed to individualise therapy. It is likely that the TSER genotype would be used in conjunction with other TYMS variants and as part of a multiple gene panel in order to better individualise therapy.

A SNP has recently been described within the second repeat of the TSER^*^3 allele, which may also affect the level of TS expression in patients by abolishing a USF1-binding site (Mandola *et al*, 2003). Preliminary *in vitro* transfection assays have shown the common allele (denoted 3RG), which occurs in 56% of three repeat alleles in Caucasians (Mandola *et al*, 2003), to have a higher translational efficiency than all other TSER alleles (Kawakami and Watanabe, 2003). In addition, a study of 208 colorectal cancer patients and 675 controls found a 1.3-fold (95% confidence intervals 0.9–1.9) increased risk of colorectal cancer for patients with the 3RG allele, implying that the polymorphism may enhance the effects of the repeat polymorphism in the TSER (Stoehlmacher *et al*, 2003). A full functional analysis of this SNP, and a large-scale study in 5FU-treated patients, is now warranted.

A third polymorphism in the TYMS gene is a 6 bp deletion located in the 3′UTR, 447 bp downstream from the stop codon (Ulrich *et al*, 2000). The deletion is present at an allele frequency of 27% in Caucasians (Lenz *et al*, 2002). Recent data suggest a significant association of the deletion allele with a reduced response to 5FU-containing chemotherapy. Patients homozygous for the presence of the 6 bp sequence had an odds ratio of 2.0 for response to 5FU-containing combination chemotherapy (McLeod *et al*, 2003). A large-scale assessment of the role of each TYMS polymorphism individually and as a haplotype is now required to determine whether prospective assessment is warranted in patients prior to 5FU-containing chemotherapy treatment.

## POLYGENIC PHARMACOGENOMIC STRATEGIES

**Figure 1:**
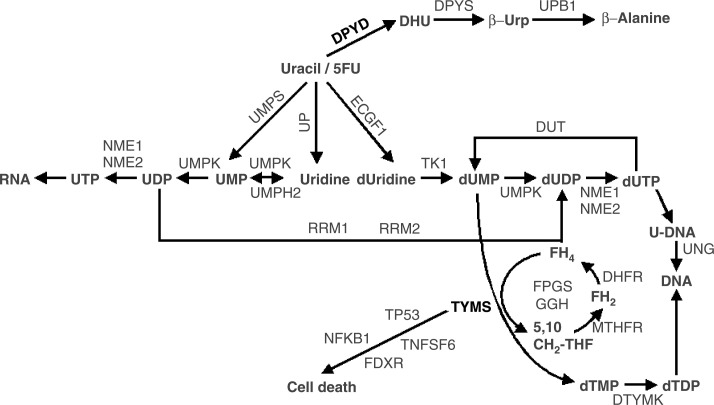
5-Fuorouracil drug pathway demonstrating the interaction of multiple gene products. Genes discussed in this review are shown in bold. The official Human Genome Organization gene nomenclature is used. Common or alternative names for each gene can be found at http://pharmacogenetics.wustl. edu.

The examples highlighted in this review demonstrate the possible utility in prescreening patients for well-characterised polymorphisms to enable the best-tolerated and most effective treatment strategies to be identified. Unfortunately, genes do not act in isolation and drugs are often involved in complex metabolic pathways in the cell before they are converted to active or inactive forms. A clear example is seen with 5-FU, which utilises the cell's pyrimidine metabolic pathway (Figure 1

). Variations in the DPYD gene can determine the amount of 5FU available for conversion to FdUMP, and variations in the TYMS gene can determine the amount of target available for inhibition by FdUMP. However, there are over 29 genes involved in the 5FU pathway (Figure 1), in which genetic variations in any or all can contribute to systemic toxicity or anti-tumour response. Initial steps are being taken to integrate drug pathway analysis, rather than single-gene studies, into clinical trials to assess the predictive power of chemotherapy activity and response. This will allow for the evaluation of gene–gene interactions in the context of anticancer drug effect. Drug pathway profiling in advance of therapy may allow us a greater chance to achieve the goal of optimising chemotherapy strategies. One can envision a future whereby comprehensive assessment of genetic variants in components of drug pathways for all approved anticancer drugs will be conducted at diagnosis. This would allow the selection of therapy that will be well tolerated and maximally efficacious. To enable this, a significant enhancement in our current understanding of the functional importance of the many genetic variants in drug pathway genes is required, in order to avoid the common finding of ‘polymorphism of unknown consequence’. Thus it is imperative that current active and planned clinical trials include correlative science components, in order to define clearly the relative contribution of pharmacogenetics to optimisation of chemotherapy.
